# Elevation of Mitochondrial Ca^2+^ Above a Plateau Level Impairs Force Production and Accelerates Fatigue in Mouse Soleus Muscle

**DOI:** 10.3390/cells15080713

**Published:** 2026-04-17

**Authors:** Joseph Bruton, Kent Jardemark

**Affiliations:** 1 Department of Physiology and Pharmacology, Karolinska Institutet, 171 77 Stockholm, Sweden; kent.jardemark@ki.se; 2 Department of Medical Biochemistry and Biophysics, Karolinska Institutet, 171 77 Stockholm, Sweden

**Keywords:** Ca^2+^, mitochondria, skeletal muscle

## Abstract

Soleus muscle fibres display modest changes in tetanic force and [Ca^2+^]_i_ during repeated contractions. In this study, we investigate whether increasing mitochondrial Ca^2+^ load during repeated contractions could induce premature fatigue. Intact, single fibres were dissected from the soleus muscles of adult mice. Mitochondrial Ca^2+^ was measured with rhod-2 in intact fibres. Fatigue was induced by 70 Hz, 350 ms tetani given at 2 s intervals in the absence and presence of 10 µM CGP-37157, a potent inhibitor of the mitochondrial Na^+^-Ca^2+^ exchanger. In soleus fibres fatigued in the absence of CGP-37157, tetanic force was significantly reduced by about 30% at the end of the fatiguing stimulation, while mitochondrial [Ca^2+^] increased to a maximum after about 50 tetani and returned to its resting level within 20 min after the end of the stimulation. In the presence of CGP-37157, the maximal mitochondrial [Ca^2+^] increase was more than twice that in control fibres. In addition, fatigue developed more rapidly and force remained depressed after the end of the stimulation. No difference in mitochondrial membrane potential or ROS production was seen between control and CGP-37157 conditions. We conclude that while modest increases in mitochondrial Ca^2^ may be beneficial, excessive mitochondrial Ca^2^ loading depresses muscle function.

## 1. Introduction

In mammalian postural muscles, fibres are rich in mitochondria with a higher density of mitochondria found closer to the sarcolemma than deeper in the fibre [1,2]. The soleus is an archetypical slow-twitch muscle where mitochondria contribute 5% to 10% of the total fibre volume, greater than that seen in type IIb fast-twitch, glycolytic muscles such as the EDL (2–3%). It should also be noted that the highest density of mitochondria is found close to the sarcolemma and the transverse tubules. Both type I and type IIa fibres have a similar arrangement of mitochondrial columns arranged across the long axis of the fibre. Type I fibres have additional prominent rows of longitudinally orientated connected mitochondria [3]. Due to the dense packing of the myofibrils and the calcium-packed sarcoplasmic reticulum, mitochondria have little room for movement. Unlike other tissues, where mitochondria have a dynamic ever-changing interconnected tubular network (mediated by members of the dynamin superfamily), mitochondria in mature skeletal muscles form relatively stable networks [4]. In addition to mitochondria clustering near the transverse tubules that conduct the action potential deep into the muscle fibre, close contacts between mitochondria, transverse tubules as well as the sarcoplasmic/endoplasmic reticulum (SR) have been described [5]. Extensive connections between the SR and the outer mitochondrial membrane with the assistance of a chaperone protein have been described in heart and other tissues. Separate distinct membrane contacts exist between the transverse tubules and mitochondria. Tunnelling or transfer of Ca^2+^ in rested, non-stimulated mitochondria has been visualised using mitochondrial-targeted indicators [6]. In muscle, mitochondria are perfectly placed to respond to action-potential induced Ca^2+^ release through the ryanodine receptors of the SR by taking up calcium via the uniporter which permits increased mitochondrial enzyme production of ATP [7,8,9]. Several mitochondrial dehydrogenases involved in the tricarboxylic acid cycle are known to require Ca^2+^ [10,11]. Training is known to enhance mitochondrial number and function and thus, to improve ATP generation in muscle and brain [12,13]. During demanding exercise, the muscles’ energy demands exceed the mitochondria’s capacity to produce ATP via oxidative phosphorylation and anaerobic respiration becomes more important. A consequence of this is accumulation of lactate, hydrogen and phosphate ions which together with an increased mitochondrial reactive oxygen species (ROS) production may contribute to the development of muscle fatigue [14,15,16]. However, whether excessive entry of calcium into mitochondria plays a role in muscle fatigue has not been examined previously.

It is well documented that mitochondria take up limited amounts of Ca^2+^ and thus modulate cytosolic-free calcium concentration ([Ca^2+^]_i_) in nerves [17], kidneys [18], and muscle [19,20]. A prolonged or uncontrolled increase in mitochondrial Ca^2+^ is considered likely to induce a decline in cellular function and accelerate senescence either directly or by increasing reactive oxygen species (ROS) generation [21,22], which in turn impairs muscle function and damage [23,24,25,26]. Mitochondria isolated from skeletal muscle after exhaustive exercise where force production is impaired contain more Ca^2+^ than those isolated from non-exercised muscle [27,28]. However, even after prolonged periods of stimulation of the nerve or muscle, mitochondrial Ca^2+^ accumulation is limited, reaching a plateau level and thus indicating perfect balance between uptake and extrusion mechanisms [20,29]. It may be speculated that this asymptotic behaviour is a protective mechanism that keeps mitochondrial Ca^2+^ below dangerous levels that would trigger excessive production of ROS or initiate apoptosis. In many tissues including nerve and muscle, extrusion of Ca^2+^ from the mitochondria is predominantly mediated by a Na^+^-Ca^2+^ exchanger that is inhibited by the benzothiazepine compound, CGP-37157 [29,30]. We hypothesise that blocking mitochondrial Na^+^-Ca^2+^ exchange during repeated tetanic contractions would result in excessively elevated levels of mitochondrial Ca2+ and thus accelerate fatigue development. We used fibres from the NMRI mouse soleus muscle because it is composed almost entirely of oxidative type I and oxidative-glycolytic type IIa fibres that are resistant to the development of fatigue due to the relative abundance of mitochondria. With abundant mitochondria to accumulate Ca^2+^, soleus fibres would provide a good model to investigate whether excessive accumulation of mitochondrial Ca^2+^ promotes faster development of fatigue.

## 2. Materials and Methods

Young (3–5 months) NMRI male mice were supplied by B & K Universal, Sollentuna, Sweden. Twenty mice were used in the experiments reported here. The studies were approved by the Swedish Ethical Review Authority (1) N117/06 Approval Date: 29 March 2006 and (2) N152/11 Approval Date: 13 May 2011. Mice were euthanized by rapid neck disarticulation, and the soleus muscles were removed. The soleus muscle in NMRI mice is composed of type I and type IIa fibres [31]. Single fibres were isolated from the muscles and mounted in a muscle bath as described previously [32]. Fibres were used only if tetanic force was greater than 350 kPa and remained stable (±5%) during the 30 min equilibration period prior to the start of the experiment.

Tetanic contractions were evoked using 70 Hz, 500 ms stimulus trains and fibres were stimulated with up to 700 tetani given at 2 s intervals. Stimulation was stopped when force fell to 40% of the initial force.

### 2.1. Solutions

Muscle fibres were superfused with a Tyrode solution containing (mM) NaCl 121, KCl 5, MgCl_2_ 0.5, Na_2_HPO_4_ 0.4, CaCl_2_ 1.8, EDTA 0.1, NaHCO_3_ 24, glucose 5.5 and foetal calf serum (0.2%, Gibco, Billings, MT, USA) and bubbled with 95% O_2_/5% CO_2_ (pH 7.4). All experiments were performed at room temperature (24–26 °C). A stock solution of 10 mM CGP-37157 (CGP, Tocris Bioscience, Bristol, UK) in DMSO was prepared and 10 µM CGP was applied for 15 min before induction of fatigue. Control fatigue runs were performed in a Tyrode solution containing 0.1% DMSO.

### 2.2. Measurement of Mitochondrial Ca^2+^, Reactive Oxygen Species, and Mitochondrial Membrane Potential Using Indicators and Laser Confocal Microscopy

To measure mitochondrial Ca^2+^, soleus fibres were incubated in 5 µM rhod-2-AM (Molecular Probes, Leiden, The Netherlands) for 90–120 min at room temperature. Rhod-2 preferentially loads into mitochondria with relatively little left in the myoplasm. It is resistant to photobleaching and gives a large increase in fluorescence upon binding to calcium [32,33]. In a separate series of experiments, soleus fibres were incubated for 10 min at room temperature with 5 µM MitoSox Red (Molecular Probes) which is a specific mitochondrial dye used to monitor mitochondrial reactive oxygen species (ROS). To measure mitochondrial membrane potential, soleus fibres were loaded with 2.6 µM rhodamine 123 (R123; Molecular Probes) for 10 min at room temperature. Following loading with the AM indicators, fibres were washed for at least 30 min. An X-Y (1024 × 1024 pixels) confocal image of the rested fibre was first obtained and fifteen min later, a second confocal image of the rested fibre in either the absence or presence of CGP was obtained and then the fibre was fatigued with a train of repeated tetanic contractions. Stimulation was paused for 6 s and images were taken after 10, 25, 50, 100, 200, 300 and 500 tetani and lastly when the stimulation was ended. During the recovery period after stimulation had ended, X-Y images were taken at regular intervals until the mitochondrial signal returned to its pre-fatigue level.

A BioRad MRC 1024 unit (Biorad Microscopy Division, Hertfordshire, UK) with a krypton/argon mixed-gas laser run at 5 mW and attached to a Nikon Diaphot 200 inverted microscope (Nikon Europe B.V. Amsterdam, The Netherlands) was used. In most experiments, a Nikon Plan Apo 40x oil immersion objective lens (N.A. 1.3, Nikon Europe B.V. Amsterdam, The Netherlands) was used. rhod-2 was excited with 568 nm light and the emitted light collected through a 585 nm long-pass filter. Both MitoSox Red and Rhodamine 123 were excited with 488 nm light and the emitted fluorescence was collected through a 522 nm bandpass filter. The minimum laser power required for acceptable images was 3% of the maximum, although up to 10% power was used. Images were stored and analysed offline with FIJI (NIH, Bethesda, MD, USA, https://hpc.nih.gov/apps/Fiji.html), accessed on 14 April 2026. No noise correction or smoothing was applied to the acquired images. To measure changes in mitochondrial fluorescence, regions of interest were defined in areas containing visible mitochondria close to the sarcolemma and the mean pixel density was calculated. Typically, at least three such regions (1000–2500 µm^2^) were measured in each fibre. Background fluorescence (the result of intrinsic fluorescence of proteins) was defined as that measured in the myoplasm which had no visible mitochondria. It should be noted that the intrinsic noise of the PMT and the rest of the acquisition system is low and is assumed to be equal in measured regions. Repeated tetanic contractions caused a clear increase in mitochondrial Rhod-2 fluorescence in soleus fibres, while the myoplasm, i.e., background intensity, showed little change. At each time point, the myoplasmic intensity was subtracted from the mitochondrial intensity. Changes in mitochondrial fluorescence intensity at each time point (F) were expressed as a ratio of that measured in the rested fibre (F_0_). This procedure allowed comparison of the mitochondrial fluorescence signal recorded in different fibres.

### 2.3. Statistics

No randomisation regarding fibre allocation to the control group or drug group was done. Experiments were done sequentially, i.e., control, drug, control, etc. All data are expressed as mean ± s.e.m. Student’s *t*-test (unpaired or paired as necessary) and a repeated-measures ANOVA (when appropriate) were used to check for statistically significant differences (Sigmaplot, 12.5 Systat Software Inc., San Jose, CA, USA). The significance level was set at *p* < 0.05. As part of the analysis process, a Sigmaplot check was done for the assumptions of the chosen statistic. Data presented here were analysed as follows: For Figures 2, 3A and 4, a one-way repeated measures ANOVA was employed. The Holm–Sidak method was used for post hoc analyses. In Figure 3B, a paired Student’s *t*-test was used and in Figure 5 an unpaired Student’s *t*-test was used.

## 3. Results

### Changes in Mitochondrial Ca^2+^ Induced by Repeated Tetani Are Increased by the Presence of CGP-37157

**Figure 1:**
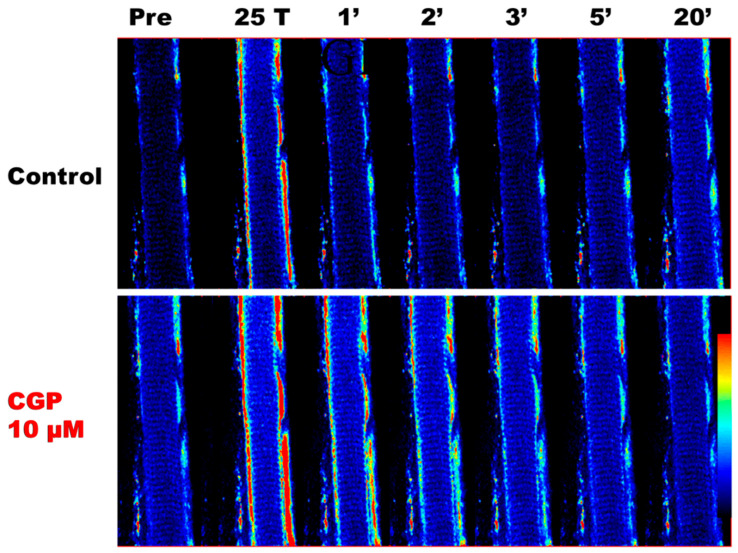
Confocal images of the changes in mitochondrial Ca^2+^ in a soleus fibre subjected to 25 tetani in Tyrode solution without (control) or with CGP-37157 (CGP). Mitochondrial rhod-2 fluorescence is shown before (Pre), after 25 tetani (25 T) and during recovery over 20 min (1′ to 20′). Mitochondrial Ca^2+^ increased more when the fibre was in the presence of CGP-37157 than in the presence of Tyrode only. In addition, the mitochondrial Ca^2+^ signal decreased more slowly after the end of stimulation in the presence of CGP-37157 than in Tyrode solution. Fibre diameter is 45 µm. Coloured bar at the bottom right indicates the level of Ca^2+^ with blue denoting low Ca^2+^ levels and red high Ca^2+^ levels.

To demonstrate that CGP-37157 could increase mitochondrial Ca^2+^ uptake, soleus fibres loaded with rhod-2 were subjected to two series of 25 tetani, the first in Tyrode only and the second series one hour later in the presence of CGP-37157. Figure 1 shows that there was an increase in the rhod-2 signal, indicating increased mitochondrial Ca^2+^, in the mitochondria close to the sarcolemma in both series of 25 tetani. This increase was more pronounced in the presence of CGP-37157 than in Tyrode. After the series of 25 tetani, the mitochondrial Ca^2+^ signal returned to the resting level more rapidly in the Tyrode solution than in the presence of CGP-37157, a further demonstration of the inhibitory effect of CGP-37157 on Ca^2+^ extrusion from the mitochondria.

**Figure 2:**
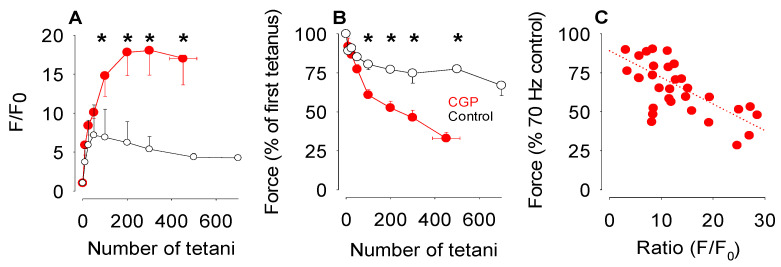
There was an increased mitochondrial Ca^2+^ and decreased tetanic force production during the induction of fatigue with repeated 70 Hz tetani. (**A**) Mean data from seven soleus fibres in the absence (open symbols) or six fibres in the presence of CGP-37157 (red filled symbols) showing the changes in the mitochondrial rhod-2 F/F_0_ during a series of up to 700 tetani. In the control fibres in the absence of CGP-37157, mitochondrial Ca^2+^ achieved a maximum after 50 tetani and thereafter declined during the remaining period of stimulation. In fibres exposed to CGP-37157, mitochondrial Ca^2+^ increased further and reached a plateau value after 200 tetani. (**B**) Tetanic force declined more rapidly in fibres exposed to CGP-37157 than in control fibres. Values in (**A**,**B**) represent mean ± s.e.m., control *n* = 7, CGP *n* = 6, * indicates statistically significant difference *p* < 0.05. (**C**) Scatterplot of tetanic force versus mitochondrial rhod-2 F/F_0_ recorded in fibres during induction of fatigue when exposed to CGP-37157, R^2^ = 0.369.

Next, soleus fibres loaded with rhod-2 were subjected to a longer series of repeated tetanic contractions to induce muscle fatigue. In control fibres fatigued in Tyrode solution, mitochondrial rhod-2 F/F_0_ reached its maximum value of 7.4 ± 3.6 after 50 tetani (Figure 2A). Thereafter, mitochondrial Ca^2+^ declined slightly throughout the rest of the series and at the end of the stimulation, mitochondrial rhod-2 F/F_0_ had fallen to 57 ± 1% of its maximum value. At the end of the stimulation, force had decreased to 66 ± 6% of initial tetanic force (Figure 2B). In soleus fibres exposed to CGP-37157, the changes in both mitochondrial Ca^2+^ and tetanic force were more pronounced. After 50 tetani, the mitochondrial rhod-2 signal was already above 10, and it continued to increase until at 300 tetani, mitochondrial rhod-2 F/F_0_ had peaked at 18.0 ± 3.2 and tetanic force had declined to 46 ± 5% (Figure 2A). In contrast to the control fibres, none of the six soleus fibres exposed to CGP-37157 were able to maintain tetanic force above the cutoff of 40% (Figure 2B) and the stimulation ended on average after 452 ± 62 tetani. When the stimulation ended, the mitochondrial rhod-2 F/F_0_ was 17.0 ± 3.4, 94% of its peak value while tetanic force had decreased to 33 ± 4% of its starting value. A scatterplot of tetanic force versus mitochondrial rhod-2 signal (Figure 2C) showed a general trend for a reduced tetanic force as the rhod-2 signal increased.

**Figure 3:**
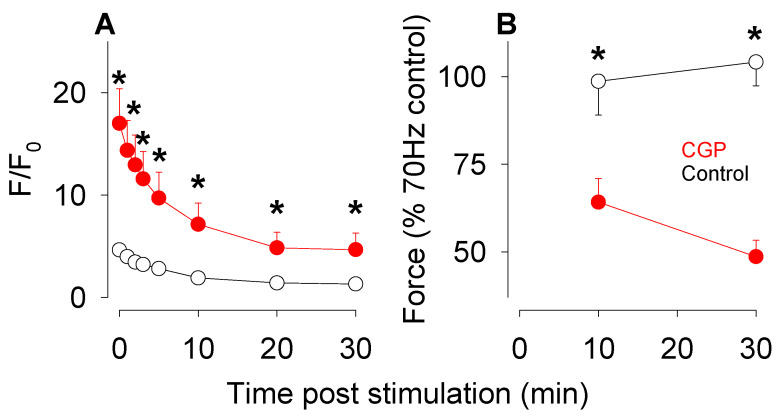
Reversal of mitochondrial Ca^2+^ changes and recovery of tetanic force following fatigue induced by repeated 70 Hz tetani. (**A**) Following the end of the stimulation protocol to induce fatigue, mitochondrial Ca^2+^ declined more rapidly in control fibres (open symbols) than in fibres exposed to CGP-37157 (red filled symbols). (**B**) Tetanic force recovered completely to its pre-fatigue in control fibres (open symbols) but not in fibres that were exposed to CGP-37157 (red filled symbols). Values represent mean ± s.e.m., control *n* = 7, CGP *n* = 6, * indicates statistically significant difference *p* < 0.05.

In control fibres after the end of the stimulation, mitochondrial rhod-2 F/F_0_ returned to its resting value within 20 min (Figure 3A). Tetanic force measured at 10 min post stimulation was 88 ± 8% and recovered completely to its resting value after 30 min (Figure 3B). The situation in CGP-37157 exposed soleus fibres was very different where mitochondrial rhod-2 F/F_0_ remained elevated (5.0 ± 1.5) even 30 min after the end of the stimulation and when tetanic force had recovered to only 48 ± 2% of its pre-fatigue value. Thus, the excessive Ca^2+^ loading of the mitochondria that occurred in the presence of CGP-37157 results in a rapid decline of force during fatigue and poor recovery of force afterwards.

**Figure 4:**
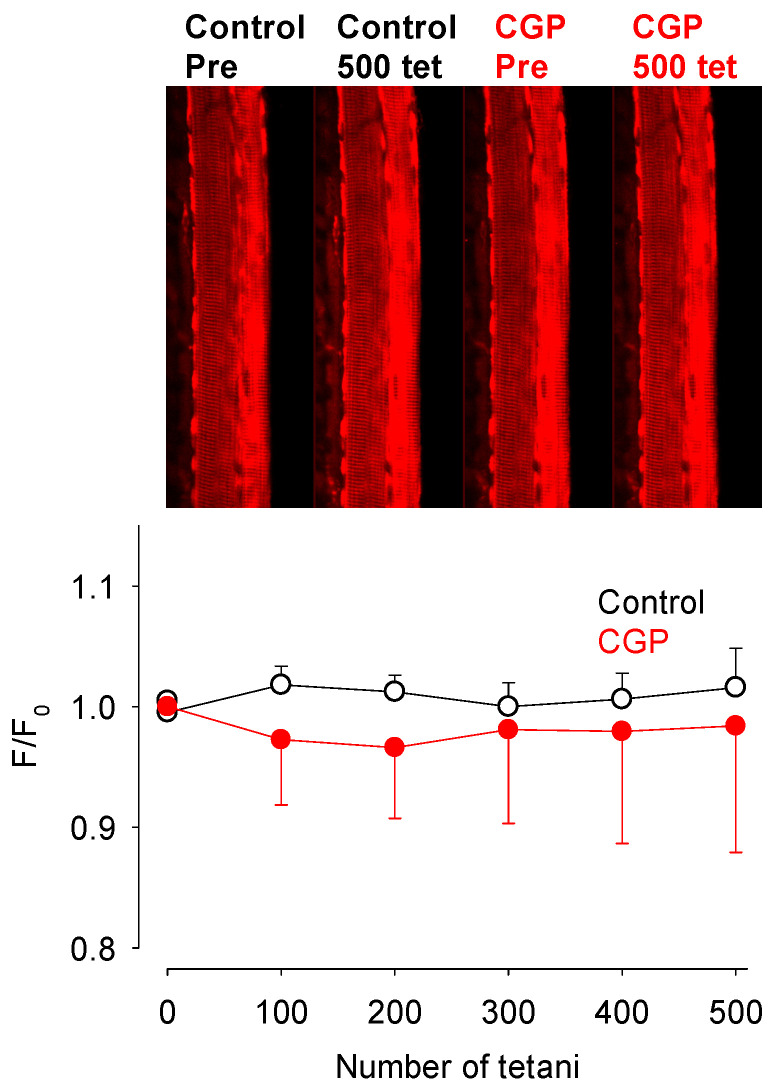
Mitochondrial membrane potential changed little during a series of repeated tetani. Top panel shows the same soleus fibre before (Pre) and at the end of a series of 500 repeated tetani (500 tet) in either Tyrode (control, open symbols) or exposed to 10 µM CGP-37157 (CGP, red filled symbols). Note that there was a 90 min recovery interval between the two series of tetani. The mitochondrial R123 showed little change in either series. Bottom panel is a graph of mean data showing that soleus mitochondrial membrane potential as measured by R123 changed little during a series of 500 repeated tetani undertaken in either Tyrode solution (control, black) or when exposed to 10 µM CGP-37157 (CGP, red). Values represent mean ± s.e.m. and *n* = 3 for both groups.

The above experiments demonstrate that repeated tetanic stimulation induced Ca^2+^ loading of the mitochondria and this Ca^2+^ load could be markedly increased with CGP-37157 present. Thus, we investigated whether this increased Ca^2+^ load had altered the mitochondrial membrane potential which would impact the mitochondrial ATP production [34]. Using the mitochondrial membrane potential probe rhodamine 123, we found that there was little change in the mitochondrial membrane potential in the soleus fibres during a series of 500 tetani in either the absence or presence of CGP-37157 (Figure 4).

**Figure 5:**
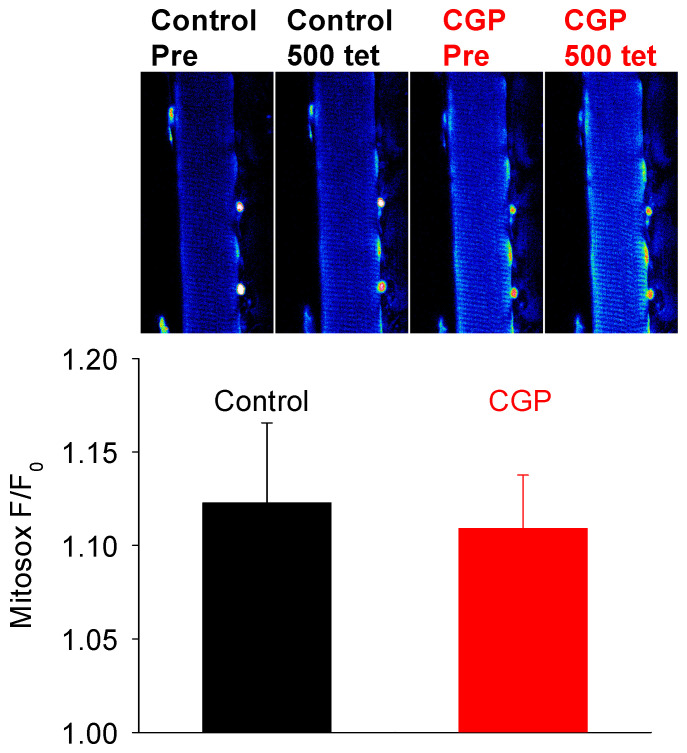
Mitochondrial ROS production in soleus fibres at the end of a series of 500 repeated tetani. Top panel shows a soleus fibre before (Pre) and at the end of a series of 500 repeated tetani (500 tet) in only Tyrode (control) or exposed to 10 µM CGP-37157 (CGP). There was a 60 min recovery interval between the two series of tetani. Bottom panel shows that mean mitochondrial ROS measured at the end of fatigue was similar in soleus fibres fatigued in Tyrode solution (control) and those exposed to 10 µM CGP-37157 (CGP). Values represent mean ± s.e.m. (control *n* = 7, CGP = 6).

Lastly, we investigated whether the greater mitochondrial Ca^2+^ loading in the presence of CGP-37157 was associated with greater levels of ROS. At the end of the fatiguing protocol, there was a modest non-significant change in ROS in the soleus fibres (Figure 5). Importantly, there was no difference in the ROS signal between the control soleus fibres and those exposed to CGP-37157. Thus, excessive Ca^2+^ loading was not associated with greater levels of ROS production in soleus fibres.

## 4. Discussion

The major results of this study show that (i) mitochondrial Ca^2+^ increased during periods of stimulation in mouse soleus fibres and this was augmented by CGP-37157 and (ii) this increased mitochondrial Ca^2+^ accumulation was accompanied by a marked impairment of contractile function. The direct stimulation of the muscle sarcolemma with brief electrical pulses allows one to be sure that these effects are happening within the muscle fibre itself and are not due to failure of transmission at the neuromuscular junction.

Removal of Ca^2+^ from the mitochondria is accomplished predominantly by a Na^+^-Ca^2+^ exchanger, which is at least ten times slower than the uniporter-mediated uptake in skeletal muscle [19,35]. Thus, significant mitochondrial Ca^2+^ uptake via the mitochondrial uniporter can occur during repeated electrical stimulation [35], such as used in the present study, due to the high [Ca^2+^]_i_ (>1 µM) achieved in each tetanus coupled with the slower extrusion of Ca^2+^ from the mitochondria. Some degree of Ca^2+^ uptake by muscle mitochondria during activity appears essential for metabolic function as mitochondrial calcium uniporter knockout mice show a reduced exercise capacity [36]. The decline in mitochondrial [Ca^2+^] that occurred after 100 tetani of the series is not due to reduced [Ca^2+^]_i_ as the Ca^2+^ transient amplitude shows no decrease during this period [32]. The decline might reflect significant buffering within the mitochondria via Ca^2+^-binding proteins such as calmitine found in the mitochondrial matrix [37,38] or cardiolipin, a negatively charged phospholipid found on the inner mitochondrial membrane [24]. It has been suggested previously that high levels of mitochondrial Ca^2+^ may bind to phosphate and form calcium phosphate compounds [29] and thus limit the free mitochondrial [Ca^2+^] in neuronal mitochondria. In the soleus fibres in the present study, the relatively rapid reversal of the mitochondrial rhod-2 signal in soleus fibres immediately after the end of stimulation suggests that the majority of the Ca^2+^ exists in the free form in the matrix of the mitochondria. On balance, it seems probable that the decline reflects increased activity of the Na^+^-Ca^2+^ exchange stimulated by the increased intracellular Na^+^ that occurs during repeated tetani [39]. CGP-37157, which inhibits the mitochondrial Na^+^-Ca^2+^ exchanger, both increased peak mitochondrial Ca^2+^ during the series of repeated tetani and slowed the rate of extrusion of Ca^2+^ after the stimulation had stopped (see Figure 2A and Figure 3A). CGP-37157 has a protective effect in neuronal excitotoxicity by blocking voltage-gated, including L-type, Ca^2+^ channels [40]. However, while L-type voltage-gated Ca^2+^ channels are present in both neurons and muscle, they differ in structure [41] and no evidence that CGP-37157 affects L-type Ca^2+^ channels in skeletal muscle has been reported (e.g., [30]).

One interesting finding of this study is the accelerated force decline observed during the induction of fatigue in the presence of CGP-37157. Tetanic force generation is governed by the amount of calcium released from the sarcoplasmic reticulum (SR). Since we demonstrate an increase in mitochondrial Ca^2+^, it can be asked if this reduces the SR-releasable Ca^2+^. Previous studies in rat cardiomyocytes [42] and skeletal muscle [43] have estimated that mitochondrial Ca^2+^ uptake is small and even during repeated contractions is not more than 1% of the total amount of SR Ca^2+^ released into the cytosol [30]. Thus, it appears unlikely that Ca^2+^ accumulation in the mitochondria can explain the decline in tetanic force.

Previously, Madsen et al. [28] reported that the elevations in mitochondrial Ca^2+^ induced by exhaustive exercise persisted for at least 60 min after the end of exercise in human muscle. In contrast, the present study finds that mitochondrial Ca^2+^ returned to control levels within 20 min of the end of repetitive activity. It should be noted that in the earlier study on human muscle, total mitochondrial Ca^2+^ was measured [28] while the rhod-2 used in the present study reports only the presence of free Ca^2+^ and not Ca^2+^ complexed with ions such as phosphate or bound to mitochondrial buffering proteins such as calmitine.

Mitochondrial Ca^2+^ uptake is implicated in both dissipation of the mitochondrial membrane potential and augmented ROS production and it is widely accepted that excessive ROS production exerts deleterious effects on cellular function [24,25,44]. Several groups have pointed out that ROS production can be considered physiologically vital as it is needed both to modify force production and modulate expression of genes involved in membranes and structural protein remodelling and metabolism [21,23,45]. Indeed, in the rat, strenuous exercise has little effect on ROS production in the soleus muscle [46]. In the present study, there were no differences in mitochondrial ROS production between soleus fibres exposed only to the Tyrode solution and those exposed to the Na^+^-Ca^2+^ exchange inhibitor, CGP-37157. This suggests that mitochondrial Ca^2+^ is of little importance in ROS generation in soleus fibres.

Little change in the mitochondrial membrane potential was observed after induction of fatigue under control conditions or exposure to CGP-37157. This is in line with results of a previous study on fast-twitch muscle fibres showing that there was no change in mitochondrial membrane potential in association with mitochondrial Ca^2+^ uptake in fast-twitch muscle fibres [20]. Thus, mitochondrial Ca^2+^ has little or no effect on mitochondrial potential possibly due to cations such as chloride accompanying Ca^2+^ entry into the mitochondria, although this needs to be confirmed by further research.

### Study Limitations

Caution must be used in extrapolating these results. First, rhod-2 is a non-ratiometric indicator and this limits our knowledge as to the exact magnitude of the increase in mitochondrial Ca^2+^. Second, the study examined only soleus fibres from NMRI mice without taking into consideration whether they were oxidative (type I) or oxidative-glycolytic (type IIb) fibres. It is not certain that glycolyic type IIb or type IIx fibres would behave the same. Third, CGP-37157 was used as a “gold-standard” inhibitor of the mitochondrial Na^+^-Ca^2+^ exchanger. However, CGP-37157 has been reported to affect other ion channels and Ca^2+^ pumps and exchangers. While resting tension and tetanic force was unchanged after the 15 min equilibration period, subtle non-specific off-target effects of CGP-37157 cannot be entirely ruled out.

## 5. Conclusions

In conclusion, during extended periods of contractile activity in soleus fibres, mitochondrial Ca^2+^ is tightly regulated and exerts a stimulatory role in mitochondrial respiration. In the presence of CGP-37157, there is increased Ca^2+^ accumulation in mitochondria which results in impaired skeletal muscle force production, although the precise mechanism remains to be elucidated.

## Data Availability

All original data presented in this study are available on request from the corresponding author.

## References

[1] Chen G., Carroll S., Racay P., Dick J., Pette D., Traub I., Vrbova G., Eggli P., Celio M., Schwaller B. (2001). Deficiency in parvalbumin increases fatigue resistance in fast-twitch muscle and upregulates mitochondria. Am. J. Physiol..

[2] Nakae Y., Stoward P.J., Shono M., Matsuzaki T. (1999). Localisation and quantification of dehydrogenase activities in single muscle fibers of *mdx* gastrocnemius. Histochem. Cell Biol..

[3] Mishra P., Varuzhanyan G., Pham A.H., Chan D.C. (2015). Mitochondrial dynamics is a distinguishing feature of skeletal muscle fiber types and regulates organellar compartmentalization. Cell Metab..

[4] Katti P., Ajayi P.T., Aponte A., Bleck C.K.E., Glancy B. (2022). Identification of evolutionarily conserved regulators of muscle mitochondrial network organization. Nat. Commun..

[5] Franzini-Armstrong C., Boncompagni S. (2011). The evolution of the mitochondria-to-calcium release units relationship in vertebrate skeletal muscles. J. Biomed. Biotechnol..

[6] Cartes-Saavedra B., Ghosh A., Hajnóczky G. (2025). The roles of mitochondria in global and local intracellular calcium signalling. Nat. Rev. Mol. Cell Biol..

[7] Dong H., Tsai S.Y. (2023). Mitochondrial properties in skeletal muscle fiber. Cells.

[8] Protasi F., Serano M., Brasile A., Pietrangelo L. (2026). Exercise protects skeletal muscle fibers from age-related dysfunctional remodeling of mitochondrial network and sarcotubular system. Cells.

[9] Rizzuto R., Pinton P., Carrington W., Fay F.S., Fogarty K.E., Lifshitz L.M., Tuft R.A., Pozzan T. (1998). Close contacts with the endoplasmic reticulum as determinants of mitochondrial Ca^2+^ responses. Science.

[10] Kavanagh N.I., Ainscow E., Brand M.D. (2000). Calcium regulation of oxidative phosphorylation in rat skeletal muscle mitochondria. Biochim. Biophys. Acta.

[11] Lee S.H., Duron H.E., Chaudhuri D. (2023). Beyond the TCA cycle: New insights into mitochondrial calcium regulation of oxidative phosphorylation. Biochem. Soc. Trans..

[12] Holloszy J.O. (1967). Biochemical adaptations in muscle. Effects of exercise on mitochondrial oxygen uptake and respiratory enzyme activity in skeletal muscle. J. Biol. Chem..

[13] Ruegsegger G.N., Vanderboom P.M., Dasari S., Klaus K.A., Kabiraj P., McCarthy C.B., Lucchinetti C.F., Nair K.S. (2019). Exercise and metformin counteract altered mitochondrial function in the insulin-resistant brain. JCI Insight.

[14] Allen D.G., Lamb G.D., Westerblad H. (2008). Skeletal muscle fatigue: Cellular mechanisms. Physiol. Rev..

[15] Constantin-Teodosiu D., Constantin D. (2021). Molecular mechanisms of muscle fatigue. Int. J. Mol. Sci..

[16] Debold E.P., Westerblad H. (2024). New insights into the cellular and molecular mechanisms of skeletal muscle fatigue: The Marion J. Siegman Award Lectureships. Am. J. Physiol..

[17] Groten C.J., MacVicar B.A. (2022). Mitochondrial Ca^2+^ uptake by the MCU facilitates pyramidal neuron excitability and metabolism during action potential firing. Commun. Biol..

[18] Wilson D.R., Arnold P.E., Burke T.J., Schrier R.W. (1984). Mitochondrial calcium accumulation and respiration in ischemic acute renal failure in the rat. Kidney Int..

[19] Sembrowich W.L., Quintinskie J.J., Li G. (1985). Calcium uptake in mitochondria from different skeletal muscle types. J. Appl. Physiol..

[20] Lännergren J., Westerblad H., Bruton J.D. (2001). Changes in mitochondrial Ca^2+^ detected with Rhod-2 in single frog and mouse skeletal muscle fibres during and after repeated tetanic contractions. J. Muscle Res. Cell Motil..

[21] Brookes P.S., Yoon Y., Robotham J.L., Anders M.W., Sheu S.S. (2004). Calcium, ATP, and ROS: A mitochondrial love-hate triangle. Am. J. Physiol..

[22] Matuz-Mares D., González-Andrade M., Araiza-Villanueva M.G., Vilchis-Landeros M.M., Vázquez-Meza H. (2022). Mitochondrial calcium: Effects of its imbalance in disease. Antioxidants.

[23] Reid M.B., Durham W.J. (2002). Generation of reactive oxygen and nitrogen species in contracting skeletal muscle. Ann. N. Y. Acad. Sci..

[24] Grijalba M.T., Vercesi A.E., Schreier S. (1999). Ca^2+^-induced increased lipid packing and domain formation in submitochondrial particles. A possible early step in the mechanism of Ca^2+^-stimulated generation of reactive oxygen species by the respiratory chain. Biochemistry.

[25] Peng T.I., Jou M.J. (2010). Oxidative stress caused by mitochondrial calcium overload. Ann. N. Y. Acad. Sci..

[26] Prylutskyy Y.I., Vereshchaka I.V., Maznychenko A.V., Bulgakova N.V., Gonchar O.O., Kyzyma O.A., Ritter U., Scharff P., Tomiak T., Nozdrenko D.M. (2017). C60 fullerene as promising therapeutic agent for correcting and preventing skeletal muscle fatigue. J. Nanobiotechnol..

[27] Duan C., Delp M.D., Hayes D.A., Delp P.D., Armstrong R.B. (1990). Rat skeletal muscle mitochondrial [Ca^2+^] and injury from downhill walking. J. Appl. Physiol..

[28] Madsen K., Ertbjerg P., Djurhuus M.S., Pedersen P.K. (1996). Calcium content and respiratory control index of skeletal muscle mitochondria during exercise and recovery. Am. J. Physiol..

[29] David G., Talbot J., Barrett E.F. (2003). Quantitative estimate of mitochondrial [Ca^2+^] in stimulated motor nerve terminals. Cell Calcium.

[30] Scorzeto M., Giacomello M., Toniolo L., Canato M., Blaauw B., Paolini C., Protasi F., Reggiani C., Stienen G.J. (2013). Mitochondrial Ca^2+^-handling in fast skeletal muscle fibers from wild type and calsequestrin-null mice. PLoS ONE.

[31] Maréchal G., Beckers-Bleukx G. (1993). Force-velocity relation and isomyosins in soleus muscles from two strains of mice (C57 and NMRI). Pflüg. Arch..

[32] Bruton J., Tavi P., Aydin J., Westerblad H., Lännergren J. (2003). Mitochondrial and myoplasmic [Ca^2+^] in single fibres from mouse limb muscles during repeated tetanic contractions. J. Physiol..

[33] Babcock D.F., Herrington J., Goodwin P.C., Park Y.B., Hille B. (1997). Mitochondrial participation in the intracellular Ca^2+^ network. J. Cell Biol..

[34] Zorova L.D., Popkov V.A., Plotnikov E.Y., Silachev D.N., Pevzner I.B., Jankauskas S.S., Babenko V.A., Zorov S.D., Balakireva A.V., Juhaszova M. (2018). Mitochondrial membrane potential. Anal. Biochem..

[35] Rizzuto R., Pinton P., Brini M., Chiesa A., Filippin L., Pozzan T. (1999). Mitochondria as biosensors of calcium microdomains. Cell Calcium.

[36] Roman B., Mastoor Y., Zhang Y., Gross D., Springer D., Liu C., Glancy B., Murphy E. (2024). Loss of mitochondrial Ca^2+^ uptake protein 3 impairs skeletal muscle calcium handling and exercise capacity. J. Physiol..

[37] Lestienne P., Bataille N., Lucas-Heron B. (1995). Role of the mitochondrial DNA and calmitine in myopathies. Biochim. Biophys. Acta.

[38] Lucas-Heron B., Le Ray B., Schmitt N. (1995). Does calmitine, a protein specific for the mitochondrial matrix of skeletal muscle, play a key role in mitochondrial function?. FEBS Lett..

[39] Juel C. (1986). Potassium and sodium shifts during in vitro isometric muscle contraction, and the time course of the ion-gradient recovery. Pflüg. Arch..

[40] Ruiz A., Alberdi E., Matute C. (2014). CGP37157, an inhibitor of the mitochondrial Na^+^/Ca^2+^ exchanger, protects neurons from excitotoxicity by blocking voltage-gated Ca^2+^ channels. Cell Death Dis..

[41] Striessnig J., Hoda J.C., Koschak A., Zaghetto F., Müllner C., Sinnegger-Brauns M.J., Wild C., Watschinger K., Trockenbacher A., Pelster G. (2004). L-type Ca^2+^ channels in Ca^2+^ channelopathies. Biochem. Biophys. Res. Commun..

[42] Andrienko T.N., Picht E., Bers D.M. (2009). Mitochondrial free calcium regulation during sarcoplasmic reticulum calcium release in rat cardiac myocytes. J. Mol. Cell. Cardiol..

[43] Baylor S.M., Hollingworth S. (2007). Simulation of Ca^2+^ movements within the sarcomere of fast-twitch mouse fibers stimulated by action potentials. J. Gen. Physiol..

[44] Suski J., Lebiedzinska M., Bonora M., Pinton P., Duszynski J., Wieckowski M.R. (2018). Relation between mitochondrial membrane potential and ROS formation. Methods Mol. Biol..

[45] Wei Y., Jia S., Ding Y., Xia S., Giunta S. (2023). Balanced basal-levels of ROS (redox-biology), and very-low-levels of pro-inflammatory cytokines (cold-inflammaging), as signaling molecules can prevent or slow-down overt-inflammaging, and the aging-associated decline of adaptive-homeostasis. Exp. Gerontol..

[46] Yoo S.Z., No M.H., Heo J.W., Park D.H., Kang J.H., Kim J.H., Seo D.Y., Han J., Jung S.J., Kwak H.B. (2019). Effects of acute exercise on mitochondrial function, dynamics, and mitophagy in rat cardiac and skeletal muscles. Int. Neurourol. J..

